# Comparative secretome analysis of rat stomach under different nutritional status

**DOI:** 10.1016/j.dib.2015.01.002

**Published:** 2015-02-13

**Authors:** Lucia L. Senin, Arturo Roca-Rivada, Cecilia Castelao, Jana Alonso, Cintia Folgueira, Felipe F. Casanueva, Maria Pardo, Luisa M. Seoane

**Affiliations:** a Grupo Fisiopatologia Endocrina, Instituto de Investigacion Sanitaria de Santiago de Compostela (IDIS), Complexo Hospitalario Universitario de Santiago (CHUS/SERGAS), Santiago de Compostela, Spain; b CIBER Fisiopatologia Obesidad y Nutricion (CB06/03), Instituto de Salud Carlos III, Santiago de Compostela, Spain; c Grupo Obesidomica, Instituto de Investigacion Sanitaria de Santiago de Compostela (IDIS), Complexo Hospitalario Universitario de Santiago (CHUS/SERGAS), Santiago de Compostela, Spain; d Laboratorio de Proteómica, Instituto de Investigación Sanitaria de Santiago de Compostela (CHUS/SERGAS), Spain; e MRC Protein Phosphorylation & Ubiquitylation Unit, University of Dundee, United Kingdom; f Department of Physiology, Research Centre of Molecular Medicine and Chronic Diseases (CIMUS), Instituto de Investigacion Sanitaria Santiago de Compostela (IDIS), Santiago de Compostela, Spain; g Laboratorio de Endocrinologia Molecular y Celular, Instituto de Investigacion Sanitaria de Santiago (IDIS), Complejo Hospitalario de Santiago (CHUS/SERGAS), Santiago de Compostela, Spain; h Department of Medicine, University of Santiago de Compostela, Spain

## Abstract

The fact that gastric surgery is at the moment the most effective treatment to fight against obesity highlights the relevance of gastric derived proteins as potential targets to treat this pathology. Taking advantage of a previously established gastric explant model for endocrine studies, the proteomic analysis of gastric secretome was performed. To validate this gastric explant system for proteomic analysis, the identification of ghrelin, a classical gastric derived peptide, was performed by MS. In addition, the differential analysis of gastric secretomes under differential nutritional status (control feeding vs fasting vs re-feeding) was performed. The MS identified proteins are showed in the present manuscript. The data supplied in this article is related to the research article entitled “Comparative secretome analysis of rat stomach under different nutritional status” [1].

**Specifications table**

**Table:** 

Subject area	*Biomedicine*
More specific subject area	*Endocrinology, Obesity*
Type of data	*Table, Figure*
How data was acquired	*2-DE and Mass spectrometry* (*4800 MALDI-TOF/TOF analyzer-Applied Biosystems,* CA)
Data format	*Raw data*
Experimental factors	*A model of gastric ex vivo tissue explant culture adapted from the previously described*[2]*was used to perform differential secretome analysis under control, fast and reefed conditions*[1].
Experimental features	*2-DE secretome map and differential analysis, MS protein identification*
Data source location	*Santiago de Compostela, Spain*
Data accessibility	*Within this article*

## Value of the data

• The optimized protocol of ex vivo gastric explants is appropriated for comparative analysis of gastric secretomes as validated by ghrelin identification by MS.
• This is the first gastric secretome comparative analysis under three different nutritional situations.
• 63 differences were statistically significant; from those 50 proteins were identified by MS. 60% of these proteins were classified as secreted [1].

## 1 Data, experimental design, materials and methods

1. *Animal models*: Adult Sprague-Dawley male rats weighing 210 g/8 weeks of age from the breading animal facilities of the University of Santiago, were housed in air-conditioned rooms (22–24 °C) under a controlled light/dark cycle (12 h light, 12 h darkness) with free access to food and water. Animals were assigned to one of three weight-matched experimental groups (*n*=10/per group): (a) control: ad libitum feeding (233.1±3.1 g); (b) fasting: the rats were food deprived for 48 h before euthanasia (191.3±3.7 g); (c) re-feeding: the animals were food deprived during 48 h, but were allowed to have free access to food 1 h before euthanasia (191.3±3.3 g).
2. *Obtaining a gastric explant system suitable for secretome proteomic analysis*: A model of ex vivo tissue explants culture adapted from the previously described [2,3] was used. Briefly, the stomach was carefully excised and transported from the animal house operating room to the laboratory in sterile KRH buffer with penicillin (100 U/ml) and streptomycin (100 μg/ml) at room temperature. Tissues were processed to eliminate any contaminant and washed thoroughly in KRH under sterile conditions in a flow laminar hood. The tissue explants of an approximate weight of 1.8 g were placed in six-well dishes (Iwaki, Tokyo, Japan) containing 4 ml of serum/phenol red free DMEM supplemented with l-glutamine (200 mM), penicillin (100 U/ml) and streptomycin (100 μg/ml). After a pre-incubation period of 1 h at 37 °C under a humidified atmosphere of 95% air-5% CO_2_, the media were aspirated, and 4 ml of fresh medium was dispensed into each well.
  Culture medium was then collected after 2 h incubation and centrifuged for 5 min at 650*g* at room temperature to remove blood cells and cell debris. Secretomes were immediately processed for sample concentration and protein purification by ultracentrifugation units (Amicon Ultra 3KDa cut off, Millipore, Billerica, USA) and TCA/acetone precipitation (2-DE Clean Up kit, GE, Uppsala, Sweden).
3. *Immunoprecipitation and identification of ghrelin to validate gastric secretomes*:
  a. *Gastric secretome processing*: secretomes were processed as previously described [1].
  b. *Immunoprecipitation*: 1 mg of total protein was incubated with 2 μg of ghrelin polyclonal antibody (Santa Cruz Biotechnology) overnight at 4 °C, followed by addition of 20 μL of 50% protein A/G-agarose beads (Santa Cruz Biotechnology) for 2 h at 4 °C. After incubation, beads were washed three times with RIPA buffer and three times with H2Omq. The pelleted beads were eluted with 0.1% TFA to be analyzed by MALDI-TOF/MS. Ghrelin MS identification is shown in Supplementary Table 1.
4. *Secretome differential analysis*:
  a. *Two-dimensional electrophoresis (2-DE) differential analysis*: Precipitated secretomes (*n*=4) were resuspended in 2-DE sample buffer containing 5 M urea, 2 M thiourea, 2 mM tributylphosphine, 65 mM DTT, 65 mM CHAPS and 0.15 M NDSB-256. Following a brief sample centrifugation to eliminate any debris, supernatant was removed and quantified (RC DC Protein Assay, BioRad Lab, CA) to prepare 300 μg of protein in a total volume of 250 μl of 2-DE sample buffer. Ampholytes were added to the sample at 0.1% servalyte 3–10, 0.05% servalyte 2–4 and 9–11 (SERVA, Heidelberg, Germany). 3–10 NL 17 cm IPG strips (BioRad, CA) were passively rehydrated in the sample and IEF was carried out in a Protean IEF cell following the manufacturer protocol (BioRad, CA). Following focusing, the IPG strips were immediately equilibrated for 30 min in 4 M urea, 2 mM thiourea, 12 mM DTT, 50 mM Tris pH 6.8, 2% SDS, 30% glycerol. The IPG strips were then placed on the top of the second dimension gels and embedded with 1% melted agarose. Proteins were separated in the second dimension on SDS-PAGE at 12% under running conditions of 45 Vh per gel, followed by 60 V per gel for 18 h using the Proteome Plus Dodeca cell (BioRad, CA), at 10 °C as constant temperature. Following electrophoresis, gels were fixed in 10% methanol: 7% acetic acid (v:v) and stained with the fluorescent dye SyproRuby (Lonza, Switzerland). Monochrome fluorescence images (16 bit) were obtained at 200 μm resolution by scanning gels with a Typhoon fluorescence scanner (GE, Uppsala, Sweden).
  b. *Image analysis*: Resulting images were submitted to Ludesi Analysis Centre (Lund, Sweden, http://www.ludesi.com) for professional image analysis using Ludesi REDFIN Solo software. This analysis allowed an optimal control over potential technical variations. Spot detection, segmentation and matching followed a strict protocol to ensure a high level of correctness. The integrated intensity of each of the spots was measured, and the background corrected and normalized. Normalization removes systematic gel intensity differences such as variations in staining, scanning time and protein loading by mathematically minimizing the median expression difference between matched spots. This allows an adequate quantification and comparison of different gels. Control feeding vs fasting vs re-feeding stomach secretomes differential image analysis showed 63 differences after applying the most restrictive statistical analysis (ANOVA, *p*<0.001, fold change 2).
  c. *In-gel digestion and mass spectrometry analysis*: Protein features from representative 2-DE gels that were chosen for mass spectrometric analysis were excised from the SyproRuby stained gels in a blue light box, and manually digested following the protocol defined by Shevchenko [4] with minor modifications: gel pieces were washed thrice with 50 mM ammonium bicarbonate in 50% methanol before a reduction step with 10 mM DTT (Sigma-Aldrich, St. Louis, MO) in 50 mM ammonium bicarbonate (Sigma-Aldrich, St. Louis, MO), followed by alkylation with 55 mM iodoacetamide (Sigma-Aldrich, St. Louis, MO) in 50 mM ammonium bicarbonate. Then, the gel pieces were rinsed with 50 mM ammonium bicarbonate in 50% methanol (HPLC grade, Scharlau, Barcelona, Spain), dehydrated by acetonitrile addition (HPLC grade, Scharlau, Barcelona, Spain) and dried in a SpeedVac (Thermo Fisher Scientific, Waltham, MA, USA). Modified porcine trypsin (Promega, Madison, WI, USA) was added to the dry gel pieces at a final concentration of 20 ng/μl in 20 mM ammonium bicarbonate; incubating them at 37 °C for 16 h. Peptides were extracted thrice by 20 min incubation in 40 μl of 60% acetonitrile in 0.5% HCOOH. The resulting peptide extracts were pooled, concentrated in a SpeedVac and stored at −20 °C. Dried samples were dissolved in 3 μl of 0.5% HCOOH. Equal volumes (0.5 μl) of peptide and matrix solution, consisted of 3 mg CHCA dissolved in 1 ml of 50% acetonitrile in 0.1% TFA, were deposited using the thin layer method, onto a 384 Opti-TOF MALDI plate (Applied Biosystems, CA). Mass spectrometric data were obtained in an automated analysis loop using 4800 MALDI-TOF/TOF analyzer (Applied Biosystems, CA). MS spectra were acquired in reflectron positive-ion mode with a Nd:YAG, 355 nm wavelength laser, averaging 1000 laser shots and at least three trypsin autolysis peaks were used as internal calibration. All MS/MS spectra were performed by selection the precursors with a relative resolution of 300 (FWHM) and metastable suppression. Automated analysis of mass data was achieved using the 4000 Series Explorer Software V3.5. MS and MSMS spectra data were combined through the GPS Explorer Software v3.6 using Mascot software v2.1. (Matrix Science) to search against a non-redundant database (SwissProt 20100326), with 30 ppm precursor tolerance, 0.35 Da MS/MS fragment tolerance and allowing one missed cleavage. All spectra and database results were manually inspected in detail using the previous software. Proteins scores greater than 41 were accepted as significant (*p*<0.05), considering positive the identification when protein score CI% (Confidence Interval) was above 98. In case of MS/MS spectra, total ion score CI% was above 95. The MS analysis identified 50 different proteins that are shown in Supplementary Tables 2 and 3 with consecutive numbers. The rank number shows the position on a representative gel illustrated in Supplementary Fig. 1.
5. *Prediction of mammalian secretory proteins*:
  Identified proteins were submitted to SecretomeP 2.0 server (Centre for Biological Sequence Analysis: http://www.cbs.dtu.dk/services/SecretomeP/), a sequence-based prediction method capable of identifying mammalian secretory proteins of the classical and non-classical secretory pathway [5,6]. For each input sequence (FASTA format) the server predicts the possibility of non-classical secretion giving high score to proteins entering the classical secretory pathway (those proteins with signal peptide). Non-classically secreted proteins should obtain an NN-score exceeding the normal threshold of 0.5. Additionally, UniprotKB Protein knowledgebase was used for protein filtering. Proteins classified as secreted are shown in Supplementary Tables 2 and 3 with consecutive numbers; also in Supplementary Table 1 and Fig. 2E from [1].

## Supplementary materials

Supplementary Data **Table 1** – **Mascot search results report of ghrelin identification in secretome from rat stomach by mass spectrometry. Supplementary Table 2** – **List of identified proteins in control feeding vs fasting vs re-feeding stomach secretomes differential analysis.** The rank indicates its position on the 2-DE reference map, the SecretomeP 2.0 the score or classification on the secretion prediction software; additionally protein name, SwissProt accession number, expected MW and pI, volume, protein score, sequence coverage, statistical significance and fold change are shown . C: Control, F: Fasting, R: Re-feeding. **Supplementary Table 3** – **Identified peptides by MS.** List of peptides for each identified protein arranged as ranked in the 2-DE reference map in Figure 1. This table contains the accession number, Mascot Score, CI, and peptide sequence(s) for each identified protein, and the Ion Score for the peptides submitted to MS/MS.

Control feeding vs fasting vs re-feeding stomach secretomes differential analysis: A representative gel image showing 63 differences after applying the most restrictive statistical analysis (ANOVA, *p*<0.001, fold change 2) is shown. Identified proteins are listed in Table 2 with consecutive numbers.
